# Screening of Active Ingredients From *Ficus tikoua* Bur. Against Rheumatoid Arthritis and Their Enrichment Using a β‐Cyclodextrin‐Based Monolith

**DOI:** 10.1002/cbdv.71871

**Published:** 2026-10-22

**Authors:** Dan Zhang, Qianru Jiang, Min Li, Hongyuan Yan, Haiyan Liu

**Affiliations:** ^1^ College of Pharmaceutical Sciences Key Laboratory of Medicinal Chemistry and Molecular Diagnosis of Ministry of Education Hebei University Baoding China

**Keywords:** active ingredients, enrichment, *Ficus tikoua* Bur, rheumatoid arthritis, screening

## Abstract

*Ficus tikoua* Bur., a perennial creeping woody liana belonging to the genus *Ficus* (Moraceae), has been traditionally used in combination therapies for rheumatoid arthritis (RA). This study aimed to screen active components from *F. tikoua* Bur. against RA. Prior to activity testing, compounds with appropriate polarity and size were enriched by a homemade β‐cyclodextrin‐based monolithic material when adjusting the polarity of the enrichment mobile phase. Anti‐inflammatory activity evaluations of different fractions were conducted using Cell Counting Kit‐8 (CCK‐8) assay and enzyme‐linked immunosorbent assay (ELISA). Results showed that the compounds in *F. tikoua* Bur. had no significant effect on RAW264.7 cell viability while effectively reducing secretion levels of inflammatory cytokines IL‐6 and TNF‐α. When a mixture of methanol and water (30:70, *v*/*v*) was used as the enrichment mobile phase, those components adsorbed on the monolith exhibited optimal inhibition effects on IL‐6 and TNF‐α production. Further separation and LC–MS analysis of the most active fraction, combined with literature data, 4‐hydroxy‐3,5‐bis(3′‐methyl‐2‐butenyl) benzoic acid‐O‐β‐D‐glucopyranoside may be the primary anti‐inflammatory constituent. Optimization of enrichment conditions revealed that the monolithic material achieved an enrichment factor of 15.352 for the screened compound.

## Graphical abstract

A β‐CD‐based monolith was prepared and used as solid‐phase extraction adsorbent for on‐line enrichment of compounds from *Ficus tikoua* Bur. Various fractions with different molecular sizes and polarities were obtained and anti‐inflammatory activity evaluations were conducted. The active ingredient against rheumatoid arthritis was screened out by further separation of the most active fraction.

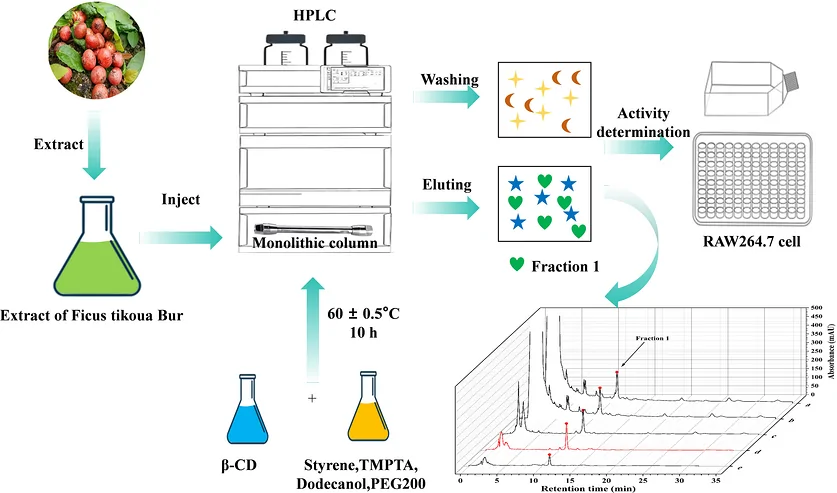

## 1 Introduction

Traditional Chinese medicine (TCM) is a gem of Chinese civilization, which utilizes natural plant, animal, and mineral‐derived substances for disease treatment. TCM has many advantages, such as simplicity, efficacy, and low side effects [1]. However, the pharmacological mechanisms of most herbal medicines remain unclear due to their complex multicomponent compositions [2], which hinders the modernization and global acceptance of TCM. *Ficus tikoua* Bur. (Moraceae), a perennial creeping woody liana of the genus *Ficus* [3], is a vital medicinal resource in traditional Chinese medicine. Its main medicinal parts are stems and leaves. This plant demonstrates therapeutic effects including heat‐clearing, dampness‐resolving, detoxification, and swelling‐reduction. It is commonly used to treat conditions such as lung‐heat cough, jaundice, infantile indigestion, rheumatic pain, amenorrhea, traumatic injuries, and nonspecific inflammatory swellings [4]. In Tujia ethnomedicine, it is classified as a wind‐cold‐dampness arthralgia herb and is used in combination to treat rheumatoid arthritis (RA) [5]. In Miao traditional medicine, fresh leaves are pounded into a paste and applied topically to reduce swelling in the early stages of mastitis.

RA, a representative disorder of systemic autoimmune dysfunction, is characterized by persistent synovial inflammation. Its classic clinical manifestations include symmetrically distributed polyarticular involvement and progressive bone erosion [6]. The global prevalence of RA ranges from 0.5% to 1.0%. Currently, RA remains incurable, with treatment strategies primarily focused on reducing inflammation, alleviating pain and morning stiffness, improving patients' symptoms, and controlling disease progression [7]. However, long‐term pharmacotherapy carries risks of multisystem adverse effects, including gastrointestinal disorders, cardiovascular complications, hepatorenal toxicity, and infection susceptibility [8]. Furthermore, the high cost and prolonged treatment duration impose significant psychological and socioeconomic burdens on patients and healthcare systems [9]. As an alternative therapy, TCM has emerged as a complementary therapeutic approach with distinct advantages, demonstrating favorable safety profile and multi‐target therapeutic efficacy [10].

Organic polymer monolithic column is a stationary phase for chromatography formed via in situ polymerization. The continuous pore structure endows it with excellent permeability and separation efficiency. Organic polymer monolithic materials demonstrate broad application prospects across various fields. When they were used as solid‐phase extraction (SPE) adsorbents, they can selectively adsorb target compounds, making them suitable for environmental [11], food safety [12], and bioanalytical applications [13].

β‐Cyclodextrin (β‐CD) is composed of seven α‐D‐glucopyranose units linked by β‐1,4‐glycosidic bonds and has a hydrophobic cavity. Owing to its host–guest inclusion properties and versatile functionalization potential, β‐CD has become an indispensable tool in pharmaceutical separation, formulation development, and analytical chemistry. Notably, β‐CD exhibits environmental compatibility due to its inherent biodegradability and low toxicity [14]. Furthermore, β‐CD derivatives demonstrate enhanced selectivity through synergistic interactions between their hydrophobic cavities and functional groups [15]. Consequently, β‐CD and its derivatives serve as an ideal platform for advanced adsorption materials via precision supramolecular design and functional modification [16, 17, 18, 19].

This purpose of this study is to isolate and screen active ingredient against RA from *F. tikoua* Bur. To achieve this goal, a β‐CD‐based monolith was prepared and used as adsorbent for SPE, which was coupled to high‐performance liquid chromatography (HPLC) to adsorb compounds from *F. tikoua*. Through this process, compounds with appropriate polarity and size were enriched and purified by adjusting the polarity of the enrichment mobile phase. Different fractions obtained through this process were screened using CCK‐8 and ELISA assays to identify those with optimal anti‐inflammatory activity. Subsequent purification of active fractions focused on isolating anti‐RA constituent, thus facilitating the discovery of therapeutic agents from *F. tikoua* Bur.

## 2 Results and Discussion

### 2.1 Characterization of Acryloyl‐β‐CD

In order to introduce the double bond into β‐CD, acryloyl‐β‐CD (A‐β‐CD) was synthesized via an esterification reaction as described in the reference [20], which is shown in Figure S1. Figure S2 presents the Fourier‐transform infrared (FTIR) spectrograms of β‐CD (a) and A‐β‐CD (b). The spectrum analysis showed a significant overlap between the characteristic C═C stretching vibration band of the acryloyl moiety and the in‐plane bending vibration absorption bands of hydroxyl groups in the β‐CD molecular framework. This molecular vibrational coupling effect limited the accurate identification of the C═C stretching vibration. However, conclusive evidence of successful acryloylation was demonstrated by the emergence of a distinct absorption band at 1727 cm^−^^1^, corresponding to the C═O stretching vibration of the esterified acryloyl group. These spectral features confirmed the successful synthesis of A‐β‐CD.

### 2.2 Optimization of β‐CD‐Based Monolithic Column

As shown in Table S1, a series of β‐CD‐based monolithic materials (H1–H9) were synthesized using A‐β‐CD and styrene as monomers, TMPTA as the crosslinker, dodecanol and PEG200 as porogenic agents, and AIBN as the initiator. The effects of monomers, crosslinker, and porogen on the permeability of the monolithic materials were systematically investigated. Compared to H2 (without A‐β‐CD), the A‐β‐CD‐based monoliths H1 and H3 exhibited significantly reduced backpressure and improved permeability. Increasing the amount of A‐β‐CD from 0.0008 g (H1) to 0.0016 g (H3) confirmed that the adding of A‐β‐CD effectively reduced flow resistance. However, when excessive A‐β‐CD was adding, mechanical strength would be compromised. Therefore, 0.0008 g of A‐β‐CD was selected to balance permeability and mechanical integrity. Reducing the content of styrene from 25 µL (H1) to 20 µL (H4), the monolithic column collapsed when it was flushed by the mobile phase. The concentration of TMPTA exhibited an inverse relationship with permeability: increasing TMPTA from 350 µL (H1) to 450 µL (H7) enhanced mechanical strength but reduced permeability. Higher content of dodecanol caused collapse (H9) indicated that porogen ratios critically influenced pore architecture. Based on comprehensive evaluation, H1 demonstrated optimal performance and was selected for subsequent studies, while H2 served as the non‐β‐CD control.

### 2.3 Characterization of β‐CD‐Based Monolithic Column

An effective adsorbent material should possess suitable pore size, higher specific surface area, and excellent mechanical properties. Figure S3 shows the scanning electron microscopy (SEM) of the monoliths with (A) and without (B) β‐CD added, both exhibited a cotton‐like porous structure. Nitrogen adsorption–desorption analysis (BET method) was employed to characterize the specific surface area and pore architecture. The β‐CD‐based monolithic material displayed a type IV isotherm (Figure S4A), which was indicative of mesoporous structure. However, the monolith without β‐CD adding exhibited a Type III isotherm (Figure S4B), which indicated the presence of macroporous or nonporous materials. Pore size distribution plots (Figure S4C,D) further demonstrated that the adding of β‐CD significantly improved the microstructural homogeneity of the monolithic material. Quantitatively, the β‐CD‐based monolithic material exhibited a specific surface area of 94.79 m^2^/g, a fourfold increase compared to the material without β‐CD added (23.89 m^2^/g). This enhancement underscored the critical role of β‐CD in expanding the accessible surface area, thereby amplifying adsorption capacity through both mesopore formation and the intrinsic host–guest interaction capabilities of cyclodextrin cavities.

As an adsorbent for online SPE, low backpressure and robust mechanical stability were critical performance indicators for monolithic materials. Figure S5A,B showed the effects of flow rates and acetonitrile concentrations in the mobile phases on column back pressures. The results showed that the monolith possessed excellent mechanical properties. Moreover, the adding of β‐CD could reduce the backpressure of the monolith, which may be due to the construction of continuous microporous channels by its cavity.

### 2.4 Effects of Chemical Constituents From *F. tikoua* Bur. on Cell Viability of Lipopolysaccharide‐Induced RAW264.7 Cells

Prior to evaluating the anti‐inflammatory activity of *F. tikoua* Bur. extract, it was essential to assess its cytotoxicity on RAW264.7 macrophages to ensure experimental validity. Cell viability was quantified using CCK‐8 method. The water‐soluble tetrazolium salt WST‐8 was enzymatically reduced by dehydrogenases to a yellow formazan product. The absorbance of this product at 450 nm correlated linearly with the number of viable cells, enabling precise quantification of cytotoxicity.

Fractions in Table S2 were reconstituted in DMEM medium supplemented with 10% fetal bovine serum (FBS) to a final concentration of 500 µg/mL and applied to RAW264.7 cells. Lipopolysaccharide (LPS) was included in the cytotoxicity assay to simulate an inflammatory microenvironment, aiming to evaluate whether the chemical constituents from *F. tikoua* Bur. exert a protective effect against LPS‐induced cellular injury, thereby providing a more accurate reflection of the potential therapeutic efficacy of *F. tikoua* Bur. against RA. As shown in Figure 1, no statistically significant differences in cell viability were observed between treatment groups and the control group. These results confirmed that the chemical constituents of *F. tikoua* Bur. extract exhibited no significant cytotoxicity toward RAW264.7 macrophages at concentrations up to 500 µg/mL. This result provided a safe dosage basis for subsequent anti‐inflammatory activity studies using 500 µg/mL as the maximum working concentration.

**FIGURE 1:**
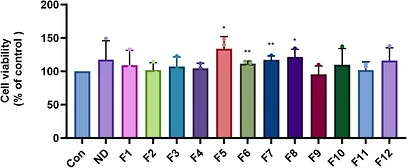
Effects of different fractions in *F. tikoua Bur*. on cell viability of LPS‐induced RAW264.7 cells.

### 2.5 Effects of Chemical Constituents From *F. tikoua* Bur. on LPS‐Induced Inflammatory Cytokines in RAW264.7 Cells

ELISA, a technique leveraging antigen‐antibody specificity and enzymatic catalysis, was employed to quantify inflammatory cytokines (TNF‐α and IL‐6) in LPS‐stimulated RAW264.7 macrophages. As shown in Figure 2, F8 significantly suppressed LPS‐induced secretion of IL‐6 and TNF‐α.

**FIGURE 2:**
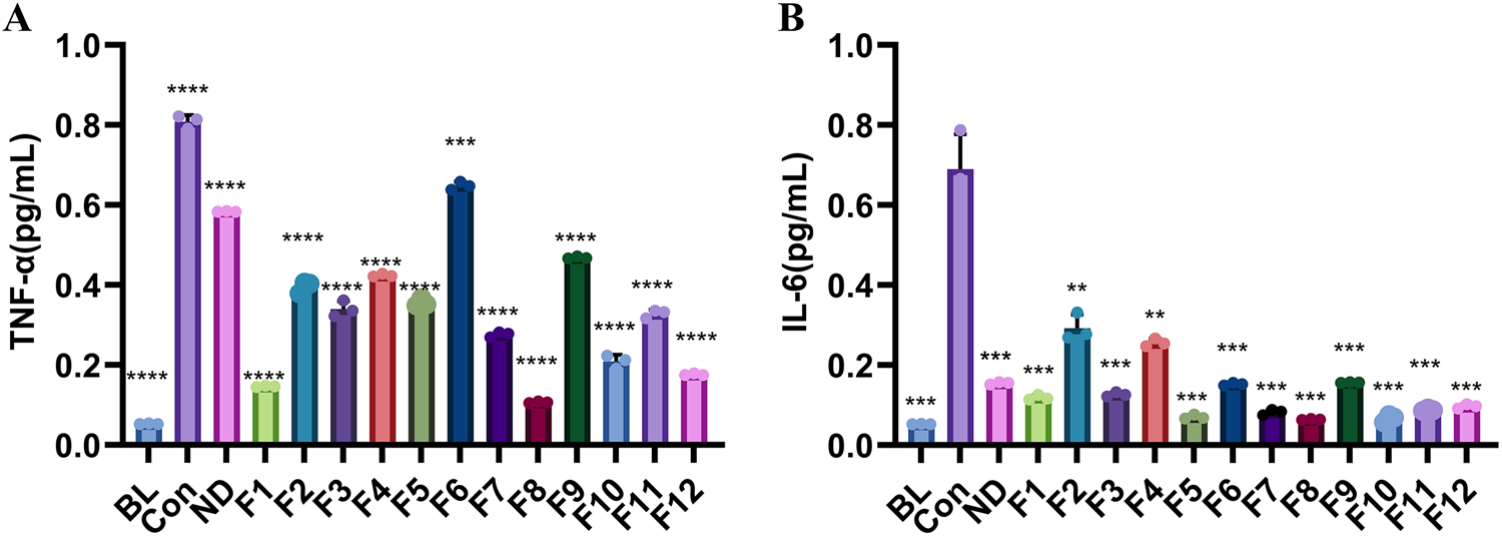
Effect of different fractions in *F. tikoua* Bur. on the expression of inflammatory cytokines TNF‐α (A) and IL‐6 (B) in LPS‐induced RAW264.7 cells.

### 2.6 Separation of Fraction F8 and Subsequent CCK‐8 and ELISA Analysis in RAW264.7 Cells

The chromatographic profile of fraction F8 separated using a reversed‐phase semi‐preparative column was shown in Figure 3A. The gradient elution conditions are shown in Table S3. Due to incomplete baseline resolution of constituents, six subfractions were collected based on retention time intervals: R1 (4‐12 min), R2 (16‐22 min), R3 (35‐45 min), R4 (61‐80 min), R5 (82‐98 min), and R6 (99‐107 min). Eluates corresponding to these intervals were collected, dried under vacuum, and reconstituted in DMEM medium (10% FBS) for cellular assays.

**FIGURE 3:**
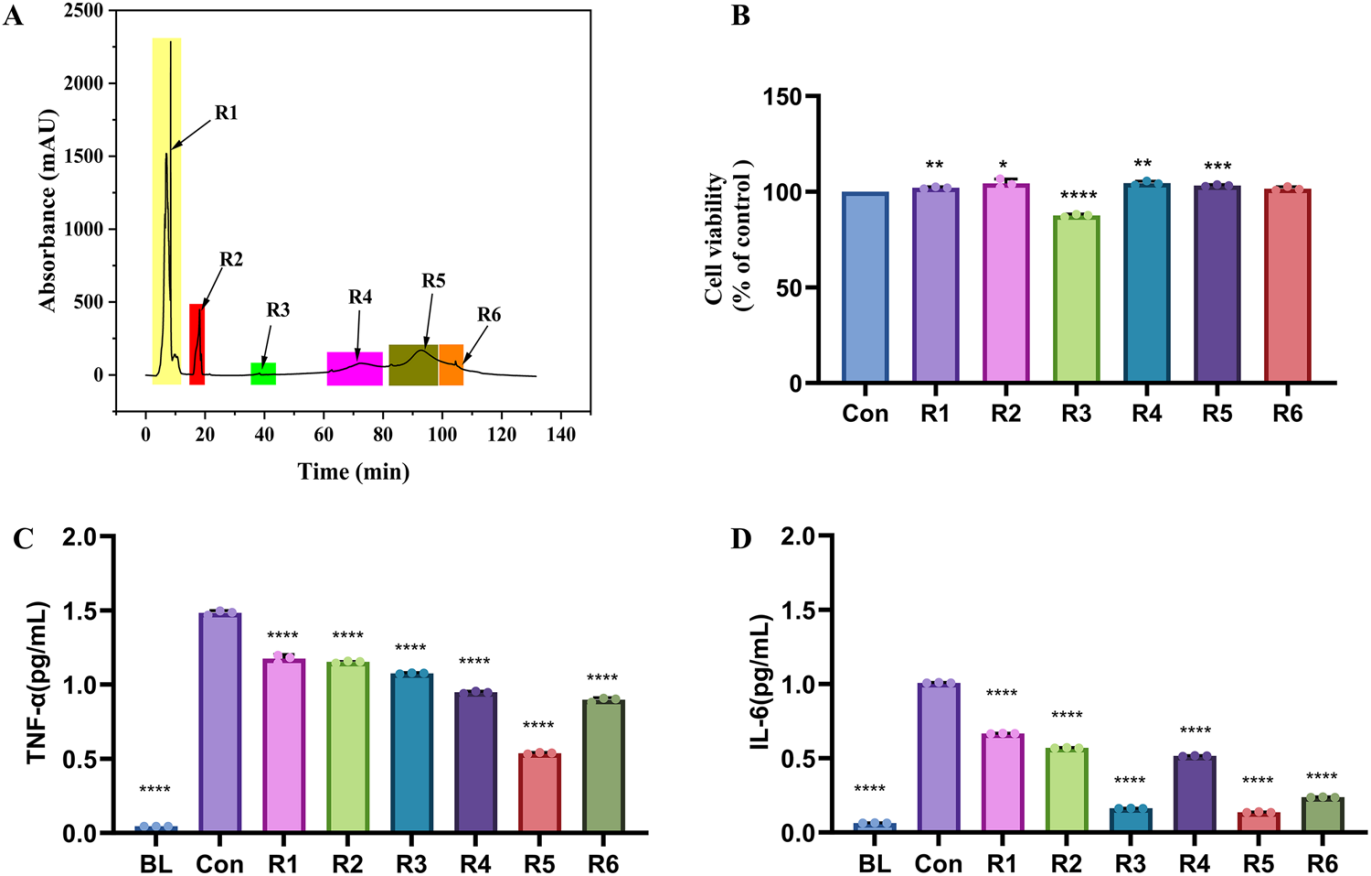
Separation profile of F8 using a reversed‐phase semi‐preparative column (A) and the effect of different parts on cell viability(B), TNF‐α (C), and IL‐6 (D) in LPS‐induced RAW264.7 cells.

The cytotoxicity of these fractions on RAW264.7 macrophages was evaluated via the CCK‐8 assay. As shown in Figure 3B, no significant cytotoxicity was observed within the concentration range of 200 µg/mL, confirming the biocompatibility of these fractions for subsequent anti‐inflammatory studies.

The effects of these fractions on the expression of inflammatory cytokines, as determined by ELISA, were shown in Figure 3C,D. Subfraction R5 exhibited significant and specific inhibitory effects on the expression of pro‐inflammatory mediators IL‐6 and TNF‐α in LPS‐induced macrophage inflammation model.

Subfraction R5 was reconstituted in 100% methanol and subjected to semi‐preparative reversed‐phase chromatography. The chromatographic profile was shown in Figure 4A. The eluate corresponding to Fraction 1 was collected, dried under vacuum, and reconstituted in DMEM medium at specified concentrations for cellular assays. Figure 4B–D showed the relations between the concentrations of Fraction 1 and the pro‐inflammatory cytokines (IL‐6, TNF‐α, and IL‐1β), which indicated that the optimal concentration was 100 µg/mL.

**FIGURE 4:**
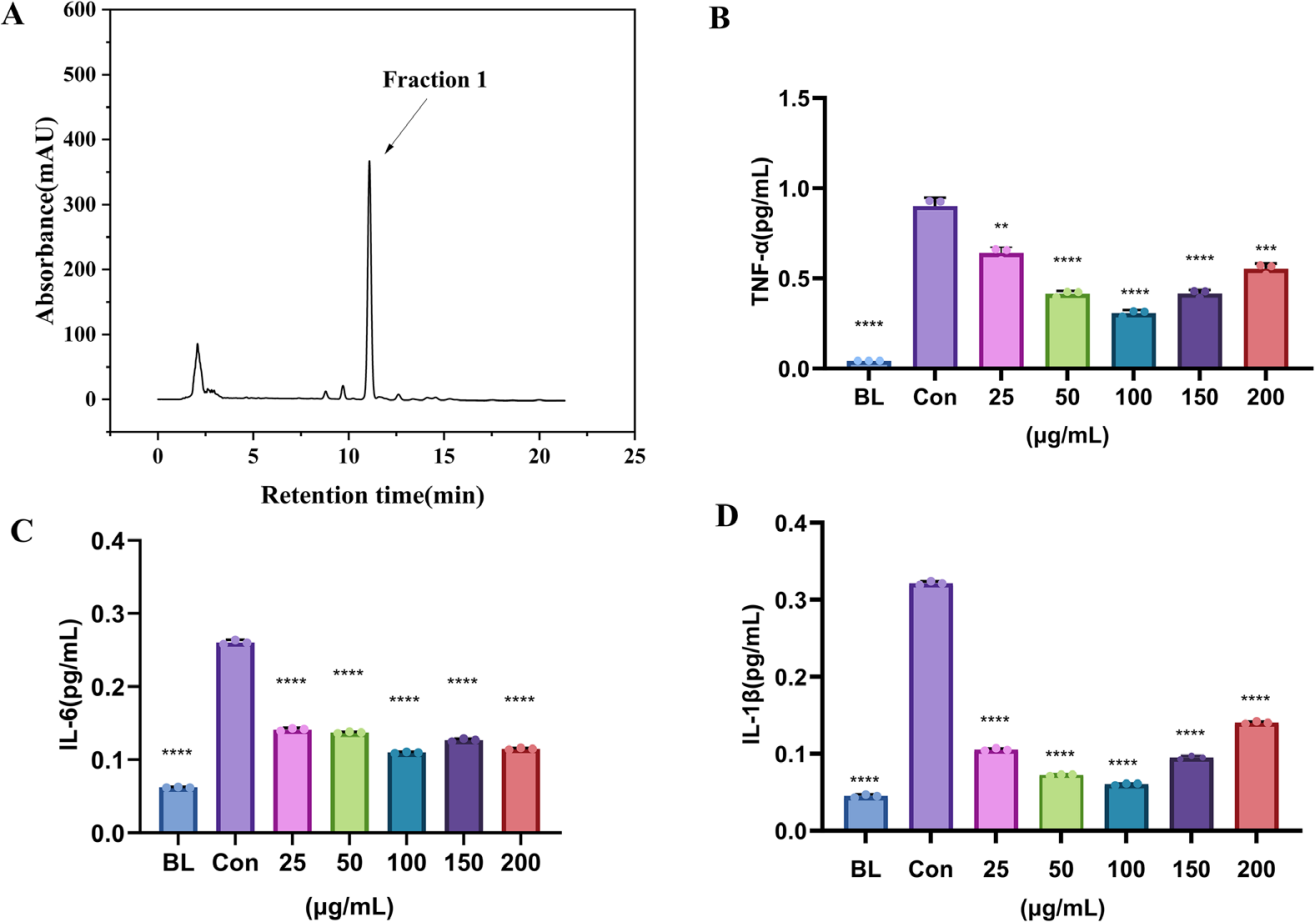
Chromatogram of R5 (A) and the effect of Fraction 1 at different concentrations on the expression of inflammatory cytokines TNF‐α (B), IL‐6 (C), and IL‐1β (D) in LPS‐induced RAW264.7 cells.

In summary, Fraction 1 exhibits promising prospects for natural anti‐inflammatory agents, although further explorations are needed, such as in vitro screen using multiple cell lines, in vivo validation, comparison with validated reference compound and establishing a molecular mechanism or therapeutic efficacy against RA.

### 2.7 Determine the Structure of Fraction 1

Fraction 1 was analyzed via liquid chromatography–mass spectrometry (LC–MS). As shown in Figure S6A, the protonated molecular ion [M + H]^+^ of Fraction 1 was detected at m/z 437.1936, which was consistent with the sodium adduct [M + Na]^+^ (m/z 459.1986) reported by Jiang et al. [21] for a known compound isolated from *F. tikoua* Bur. So Fraction 1 may be 4‐hydroxy‐3,5‐bis(3′‐methyl‐2‐butenyl) benzoic acid‐O‐β‐D‐glucopyranoside, and its structural formula was shown in Figure S6B (C_23_H_32_O_8_).

In order to further determine the structure of Fraction 1, Molisch reaction was carried out. A characteristic purple ring was observed at the phase boundary, confirming the presence of a glycosidic structure in Fraction 1.

### 2.8 Enrichment Ability of β‐CD‐Based Monolithic Column for Fraction 1

In order to investigate the enrichment ability of β‐CD‐based monolithic material for Fraction 1, the enrichment mobile phase compositions were systematically evaluated. When the extract of Ficus tikoua Bur. was injected into the monolith, non‐adsorbed fractions were washed out by different enrichment mobile phases. Then the components being adsorbed on the monolith were eluted using acetonitrile. The eluates were collected and separated on a C18 analytical column. As shown in Figure 5A, not only highest enrichment efficiency for Fraction 1 but also superior impurity removal was obtained when the enrichment mobile phase was composed of acetonitrile and water (60:40, *v*/*v*) (Figure 5A(d)).

**FIGURE 5:**
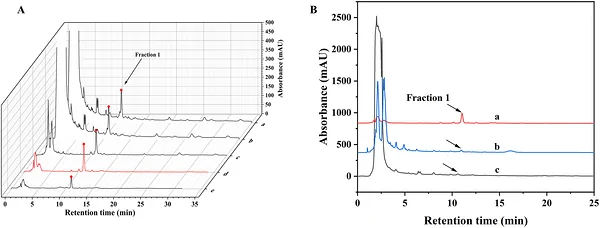
Effect of the composition of enriched mobile phases (A) and type of monolithic column (B) on the enrichment of Fraction 1 and impurities removal ability. (A): the enrichment mobile phase was a mixture of acetonitrile‐water (*v*/*v*), (a) 30:70; (b) 40:60; (c) 50:50; (d) 60:40; (e) 70:30. (B): chromatograms of the extracts of *Ficus tikoua* Bur. after (a and b) and before (c) treated by the monolithic column. The monoliths used for (a) and (b) refered to the column H1 and H2 listed in Table S1, respectively. Chromatographic conditions: C18 analytical column (25 cm × 4.6 mm i.d.); mobile phase, acetonitrile/water (55:45, v/v), flow rate, 1 mL/min; column temperature, 30°C; detection wavelength: 214 nm (UV–vis detector).

Figure 5B showed the effect of β‐CD on the enrichment ability of the monolith for Fraction 1, which demonstrated that the monolithic material with β‐CD (H1) exhibited significantly enhanced enrichment capacity for Fraction 1 and more effective impurity removal compared to that of H2 (without β‐CD). This superior performance may be attributed to the synergistic effects of β‐CD's hydrophobic cavity and hydrophilic hydroxyl groups located on the external surface, which enabled it to form multiple forces with Fraction 1, such as hydrophobic interactions, hydrogen bonding, and van der Waals interactions. After calculation, enrichment factor for Fraction 1 was 15.352.

## 3 Conclusions

This study systematically investigated the bioactive constituents of *F. tikoua* Bur. with potential anti‐RA activity. Using the self‐made β‐CD‐based monolith as the adsorbent for SPE, the chemical constituents in *F. tikoua* Bur. can be quickly enriched and separated into different parts according to their polarities and size by adjusting the mobile phase composition. By activity tracking and further separation, a compound was identified, which exhibited certain anti‐inflammatory activity.

## 4 Experimental Section

### 4.1 Instruments

The TriStar II 3020 surface area and porosity analyzer was manufactured by Micromeritics Instrument Corporation (USA). The FTIR‐850 FTIR spectrometer was manufactured by Tianjin Gangdong Sci. & Tech. Co., Ltd. (Tianjin, China). The DZF‐6020 vacuum drying oven was manufactured by Shanghai Boxun Industrial Co., Ltd. (Shanghai, China). The UPT‐II‐5T ultrapure water system by Chengdu Chaochun Technology Co., Ltd. (Chengdu, China). The Thermo Unitmate 3000 HPLC system and LC–MS (UltiMate 3000 RSLC + Q Exactive) were purchased from Thermo Fisher Scientific Inc. (USA). The KQ‐500DE ultrasonic cleaner by Jiangsu Kunshan Chemical Instrument Co., Ltd. (Jiangsu, China); the microplate reader by Zhuodi Instrument Equipment Co., Ltd. The OZKW‐4 electronic constant temperature water bath by Beijing Zhongxing Weiye Instrument Co., Ltd. (Beijing, China). The −80°C ultra‐low temperature freezer by Beijing Jinfu Renhao Technology Development Co., Ltd. (Beijing, China). The BT25S electronic analytical balance by Sartorius AG (Germany); the JSM‐7500 cold‐field emission scanning electron microscope by JEOL Ltd. (Japan). The clean bench and CO_2_ cell culture incubator by Zhejiang Fuxia Medical Technology Co., Ltd. (Zhejiang, China). The vacuum freeze dryer by Hunan Xiangyi Boyikang Biotechnology Co., Ltd. (Hunan, China).

### 4.2 Reagents and Materials

Methanol and acetonitrile (HPLC grade) were purchased from Shanghai Xingke High Purity Reagent Co., Ltd. (Shanghai, China). *F. tikoua* Bur. was gathered from Yibin, Sichuan Province, China, in April 11, 2024. The plant was identified by Prof. Huaizhong Guo, a researcher at the College of Pharmaceutical Sciences, Hebei University. A voucher specimen (20240411) has been deposited at the Key Laboratory of Medicinal Chemistry and Molecular Diagnosis of Ministry of Education, Hebei University. IL‐6 and TNF‐α ELISA kits, DMEM medium, and CCK‐8 assay kits were supplied by Jianglai Biotechnology Co., Ltd. Trimethylolpropane triacrylate (TMPTA) was acquired from Shanghai Aladdin Reagent Co., Ltd. (Shanghai, China). Dodecanol (C_12_OH) and *n*‐butanol (*n*‐BuOH) were sourced from Tianjin Comio Chemical Reagent Co., Ltd. (Tianjin, China). β‐CD and acryloyl chloride were procured from Tianjin Xiensi Biochemical Technology Co., Ltd. (Tianjin, China). Styrene was provided by Tianjin Damao Chemical Reagent Factory (Tianjin, China). Azobisisobutyronitrile (AIBN) was obtained from Tianjin Guangfu Fine Chemical Research Institute (Tianjin, China). ABTS and DPPH were purchased from Wuhan Kenuosi Technology Co., Ltd. (Wuhan, China). Mass spectrometry‐grade methanol was supplied by Anhui Zesheng Technology Co., Ltd. (Anhui, China). α‐Naphthol was acquired from Aladdin Biochemical Technology Co., Ltd. Concentrated sulfuric acid and anhydrous ethanol were obtained from Tianjin Damao Chemical Reagent Factory (Tianjin, China) and Tianjin Huihang Chemical Technology Co., Ltd. (Tianjin, China), respectively. All solutions were filtered through 0.45 µm membranes prior to use. Raw 264.7 (RRID: CVCL_0493): Mouse Monocytic Macrophage Leukemia Cell Line, which was obtained from the Chinese Academy of Sciences.

### 4.3 Preparation of β‐CD Based Monolithic Column

#### 4.3.1 Synthesis of Acryloyl‐β‐CD

The synthesis of A‐β‐CD was conducted as follows: in a 200 mL round‐bottom flask, 1 mmol β‐CD was dissolved in 30 mL of *N*,*N*‐dimethylformamide (DMF). After 1.17 mL triethylamine being added, the mixture was sonicated for 10 min. The flask was then placed in an ice bath, and 1.5 mL acryloyl chloride was slowly added dropwise. The reaction was allowed to proceed for 30 min under ice‐cold conditions, followed by an additional 30 min at room temperature. A white precipitate formed upon standing. The precipitate was removed by vacuum filtration, and the filtrate was collected. Twice the volume of acetonitrile was added to the filtrate, which resulted in the precipitation of crude A‐β‐CD. The crude product was vacuum‐filtered and washed 2–4 times with acetonitrile. The resulting solid was dried under vacuum at 80°C for 12 h.

#### 4.3.2 Synthesis of β‐CD‐Based Monolithic Column

The monolithic material was synthesized as follows: A‐β‐CD, styrene, TMPTA, dodecanol, polyethylene glycol 200 (PEG200), and AIBN were sequentially added to a 10 mL clean centrifuge tube. The mixture was vortexed thoroughly and sonicated for 45 min under vacuum to ensure complete dissolution of A‐β‐CD and AIBN. The homogeneous solution was transferred into a pre‐sealed stainless‐steel column (200 mm × 4.6 mm i.d.) and the other end was sealed. The column was then immersed in a thermostatically controlled water bath at 60°C ± 0.5°C for 10 h. After completion of the reaction, the β‐CD‐based monolithic column was connected to a HPLC system and flushed with methanol at a flow rate of 1.0 mL/min for 90 min to remove residual porogenic agents.

### 4.4 Preparation of Actual Sample

*F. tikoua* Bur. was pulverized and sieved to obtain homogeneous particles. Five grams of powder was mixed with 100 mL of 95% ethanol and subjected to reflux extraction at 80°C for 2 h. The resulting mixture was vacuum‐filtered and the filtrate was concentrated via rotary evaporation to remove the solvent. The residue was reconstituted in 10 mL of methanol and filtered through a 0.22 µm membrane to yield the extract. The final extract was stored at 4°C for subsequent experiments.

### 4.5 Enrichment and Separation of Chemical Components in *F. tikoua* Bur

The prepared β‐CD‐based monolithic column was integrated into the HPLC system. After equilibration with different enrichment mobile phases, the extract of *F. tikoua* Bur. was injected for selective adsorption of specific constituents. Non‐adsorbed fractions were washed and collected, followed by solvent removal under a nitrogen stream, which were labeled as F1, F3, and so forth. The components being adsorbed on the monolith were subsequently eluted using methanol/water (90:10, *v*/*v*) as the mobile phase, and the eluates were collected and dried, labeled as F2, F4, and so forth. The relationship between enrichment mobile phases and collected fractions was summarized in Table S2.

Chromatographic conditions: monolithic column, 200 mm × 4.6 mm i.d.; flow rate, 1 mL/min; column temperature, 30°C; detection wavelength: 214 nm (UV–vis detector).

### 4.6 Cell Culture

RAW264.7 cells were cultured in Dulbecco's modified Eagle's medium supplemented with 10% FBS.

### 4.7 Evaluation of Cytotoxicity of Different Fractions

To systematically assess the cytotoxicity and bioactivity of fractions listed in Table S2, CCK‐8 method was performed under standardized conditions, with experimental controls to eliminate nonspecific interference.

Cell viability (%) was calculated using following formula:

$$\mathrm{Cell}\;\mathrm{viability}\;\left(\%\right)=\frac{A1-A2}{A3-A2}\times 100\%$$

where: *A*1: OD of untreated cells with CCK‐8 reagent, *A*2​: OD of wells with DMEM + 10% FBS and CCK‐8 reagent (no cells), *A3*​: OD of wells containing cells, CCK‐8 reagent, and test fraction.

RAW 264.7 macrophages were pretreated with various fractions for 2 h, followed by continuous stimulation with LPS at a concentration of 200 ng/mL for 24 h. After the designated culture period, cell culture supernatants were collected, and the levels of inflammatory cytokines were quantitatively determined using a double‐antibody sandwich ELISA.

### 4.8 Effect of Different Fractions on Inflammatory Mediator Production

The anti‐inflammatory effects of fractions on inflammatory mediator production were quantitatively assessed by ELISA, with particular emphasis on interleukin‐6 (IL‐6) and tumor necrosis factor‐alpha (TNF‐α).

### 4.9 LC–MS Analysis of Fraction 1

LC–MS conditions: C18 column (150 mm × 4.6 mm, Thermo Scientific); column temperature: 30°C; injection volume: 1 µL; flow rate: 0.3 mL/min; mobile phase: acetonitrile/water (55:45, *v*/*v*); ESI positive ion mode; normalized collision energy (NCE): 35%.

### 4.10 Enrichment Factor

Enrichment factor was calculated by following equation:

 $\mathrm{EF}=C_{SPE}/C_{0}$where *C*_SPE_ and *C*_0_ were the concentrations of Fraction 1 in the eluate after SPE and the original sample, respectively.

### 4.11 Statistical Analyses

All data were expressed as mean ± SD. Each experiment was performed with three independent replicates. Statistical analysis was performed using GraphPad Prism. Comparisons among multiple groups were conducted using one‐way analysis of variance (ANOVA), followed by Tukey's post‐hoc test for multiple comparisons. Statistical significance was set at *p* < 0.05 (*p* > 0.05; * *p *< 0.05; ** *p *< 0.01; *** *p *< 0.001; **** *p* < 0.0001).

## Author Contributions

**Dan Zhang**: investigation, methodology, and writing – original draft. **Qianru Jiang**: investigation. **Min Li**: investigation. **Hongyuan Yan**: conceptualization. **Haiyan Liu**: conceptualization, writing – review and editing, and funding acquisition. All authors have reviewed and agreed to publish the manuscript.

## Funding

The work was supported by the National Natural Science Foundation of China (no. 21974034).

## Conflicts of Interest

The authors declare no conflicts of interest.

## Supporting information




**Supporting File 1**: cbdv71871‐sup‐0001‐SuppMat.docx.

## Data Availability

The authors have nothing to report.

## References

[1] L. Qiao , M. Han , S. Gao , et al., “Research Progress on Nanotechnology for Delivery of Active Ingredients From Traditional Chinese Medicines,” Journal of Materials Chemistry B 8, no. 30 (2020): 6333–6351, 10.1039/d0tb01260b. 32633311

[2] K. Wang , Q. Chen , Y. Shao , et al., “Anticancer Activities of TCM and Their Active Components Against Tumor Metastasis,” Biomedicine & Pharmacotherapy 133 (2021): 111044, 10.1016/j.biopha.2020.111044. 33378952

[3] Y. Li , X. Yan , J. Hu , et al., “Appearance Quality, Nutritional Value, and Aroma Components of Wild Diguo (*Ficus tikou* Bur.) Fruit Collected From Southwest China,” Food Science & Nutrition 12, no. 6 (2024): 4399–4407, 10.1002/fsn3.4106. PMC11167135 38873439

[4] J. Wang , D. Shi , Y. Bai , and Y. Liu , “High‐Throughput Sequencing Uncover *Ficus Tikoua* Bur. Chloroplast Genome,” Journal of Plant Biochemistry and Biotechnology 29, no. 2 (2020): 171–182, 10.1007/s13562-019-00537-9.

[5] Y. Gong , W. Luo , H. Chen , B. Ren , W. Hu , and L. Li , “Systematical Ingredient Investigations of *Ficus tikoua* Bur. Fruit and Immunoregulatory and Antioxidant Effects of Different Fractions,” Molecules 27, no. 20 (2022): 6880, 10.3390/molecules27206880. PMC9608957 36296474

[6] A. V. Bervinova , V. A. Palikov , E. S. Mikhailov , et al., “Efficacy of *Ficus tikoua* Bur. Extract in Ethylene Glycol‐Induced Urolithiasis Model in SD Rats,” Frontiers in Pharmacology 13 (2022): 974947, 10.3389/fphar.2022.974947. PMC9464938 36105205

[7] K. Bhamidipati and K. Wei , “Precision Medicine in Rheumatoid Arthritis,” Best Practice & Research in Clinical Rheumatology 36, no. 1 (2022): 101742, 10.1016/j.berh.2022.101742. PMC8977251 35248489

[8] K. D. Deane and V. M. Holers , “Rheumatoid Arthritis Pathogenesis, Prediction, and Prevention: An Emerging Paradigm Shift,” Arthritis & Rheumatology 73, no. 2 (2021): 181–193, 10.1002/art.41417. PMC7772259 32602263

[9] L. R. Espinoza and I. García‐Valladares , “Microbios y Articulaciones: La Relación Entre Infección y Articulaciones,” Reumatologia Clinica 9, no. 4 (2013): 229–238, 10.1016/j.reuma.2012.06.008. 22944142

[10] F. Axel , G. Benoît , H. Bridget , et al., “Global Epidemiology of Rheumatoid Arthritis,” Nature Reviews Rheumatology 18, no. 10 (2022): 591–602, 10.1038/s41584-022-00827-y. 36068354

[11] L. P. Jiang , N. Li , L. Q. Liu , X. Zheng , F. Y. Du , and G. H. Ruan , “Preparation and Application of Polymerized High Internal Phase Emulsion Monoliths for the Preconcentration and Determination of Malachite Green and Leuco‐Malachite Green in Water Samples,” Journal of Analysis and Testing 4 (2020): 264–272, 10.1007/s41664-020-00145-w.

[12] F. Wu , L. Li , P. Guan , K. Tang , S. Yu , and C. F. Ding , “Simultaneous Enrichment and Analysis of Benzimidazole by In‐Tube SPME‐MS Based on Poly (3‐Acrylamidophenylboronic Acid‐Co‐Divinylbenzene‐co‐N,N'‐Methylenebi‐ Sacrylamide) Monolithic Column,” Talanta 224 (2021): 121402, 10.1016/j.talanta.2020.121402. 33378997

[13] O. Mompó‐Roselló , M. Vergara‐Barberán , E. F. Simó‐Alfonso , and J. M. Herrero‐Martínez , “In Syringe Hybrid Monoliths Modified With Gold Nanopar‐ticles for Selective Extraction of Glutathione in Biological Fluids Prior Toits Determination by HPLC,” Talanta 209 (2020): 120566, 10.1016/j.talanta.2019.120566. 31892018

[14] F. M. Bezerra , M. J. Lis , H. B. Firmino , et al., “The Role of β‐Cyclodextrin in the Textile Industry—Review,” Molecules 25, no. 16 (2020): 3624, 10.3390/molecules25163624. PMC7465207 32784931

[15] S. Angelova , S. Pereva , T. Dudev , and T. Spassov , “Cyclodextrins' Internal Cavity Hydration: Insights From Theory and Experiment,” Inorganics 13, no. 1 (2025): 28, 10.3390/inorganics13010028.

[16] E. D. Ozelcaglayan and W. J. Parker , “β‐Cyclodextrin Functionalized Adsorbents for Removal of Organic Micropollutants From Water,” Chemosphere 320 (2023): 137964, 10.1016/j.chemosphere.2023.137964. 36736473

[17] Y. Zhang , J. Wang , F. Liu , L. Wang , and G. Gao , “Preparation of Cylindrical Chitosan/β‐Cyclodextrin/MIL‐68(Al) Foam Column for Solid‐Phase Extraction of Sulfonamides in Water, Urine, and Milk,” Journal of Chromatography A 1730 (2024): 465130, 10.1016/j.chroma.2024.465130. 38955130

[18] Y. Miyah , N. El Messaoudi , M. Benjelloun , et al., “A Comprehensive Review of β‐Cyclodextrin Polymer Nanocomposites Exploration for Heavy Metal Removal From Wastewater,” Carbohydrate Polymers 350 (2025): 122981, 10.1016/j.carbpol.2024.122981. 39647935

[19] L. X. Chuin , S. Kamaruzaman , S. M. Praveena , and N. Yahaya , “Recent Applications of β‐Cyclodextrin in Selective Adsorption of Pesticides, Heavy Metals, and Organic Pollutants From Water Samples: Mini Review,” Microchemical Journal 206 (2024): 111583, 10.1016/j.microc.2024.111583.

[20] H. X. Chen , B. Sun , and L. Chen , “A Preparation Method and Application of a β‐cyclodextrin Hybrid Polymer Monolithic Microcolumn”, CN, 2016110492105 [P]. 2019‐05‐28.

[21] Z. Y. Jiang , S. Y. Li , W. J. Li , et al., “Phenolic Glycosides From *Ficus tikoua* and Their Cytotoxic Activities,” Carbohydrate Research 382 (2013): 19–24, 10.1016/j.carres.2013.09.008. 24157282

