# Supplementary material for: Screening of Active Ingredients From Ficus tikoua Bur. Against Rheumatoid Arthritis and Their Enrichment Using a β‐Cyclodextrin‐Based Monolith
Source: Chem Biodivers. 2026 Oct 22;23(10):e71871. doi: 10.1002/cbdv.71871 (PMC13691381; doi:10.1002/cbdv.71871)
Supplement: Supplementary file 1 [file CBDV-23-e71871-s001.docx]

Figure S1 Synthesis of A-β-CD

Figure S2 FT-IR spectrograms of β-CD (a) and A-β-CD (b)

Figure S3 SEM images of the monoliths with (A) and without (B) β-CD added

Figure S4 Nitrogen sorption isotherms and corresponding pore size distribution profiles of monolithic materials with (A and C) and without (B and D) β-CD added

Figure S5 Effects of acetonitrile concentrations (A) and flow rates (B) on column back pressures

(a) β-CD based monolithic column

(b) organic polymer monolithic column without β-CD added

Figure S6 Protonated molecular ion [M+H]⁺ (A) and structural formula‌ (B) of Fraction 1

|  | **A-β-CD (mg)** | **Styrene**  **(μL)** | **TMPTA(μL)** | **Dodecanol** **(μL)** | **PEG200 (μL)** | **AIBN (g)** | **Back pressure^a^**  **(MPa)** | **Permeability^b^**  **(K_0_, 10^-14^m^2^)** |
| --- | --- | --- | --- | --- | --- | --- | --- | --- |
| H1 | 0.8 | 25 | 350 | 925 | 50 | 0.004 | 0.7 | 2.51 |
| H2 | 0 | 25 | 350 | 925 | 50 | 0.004 | 1.3 | 1.72 |
| H3 | 1.6 | 25 | 350 | 925 | 50 | 0.004 | 0.6 | 2.62 |
| H4 | 0.8 | 20 | 350 | 925 | 50 | 0.004 | - | - |
| H5 | 0.8 | 30 | 350 | 925 | 50 | 0.004 | 1.1 | 1.83 |
| H6 | 0.8 | 25 | 300 | 925 | 50 | 0.004 | 0.8 | 2.65 |
| H7 | 0.8 | 25 | 400 | 925 | 50 | 0.004 | 1.7 | 1.17 |
| H8 | 0.8 | 25 | 375 | 850 | 50 | 0.004 | 1.0 | 1.95 |
| H9 | 0.8 | 25 | 325 | 1000 | 50 | 0.004 | - | - |

TABLE S1 Optimization of β-CD based monolithic column

^a^Back pressure was measured under 100% methanol as mobile phase at a flow rate of 1.0 mL/min.
^b^Permeability (K_0_) was calculated via Darcy's law:K_0_=FηL(πr^2^ΔP)^-1^

where:

*F*: Flow rate (1.0mL/min)

*η*: Dynamic viscosity of methanol (0.37×10^−3^Pa s)

*L*: Effective column length (20cm)

*r*: Inner radius of monolithic material (0.23cm)

Δ*P*: Pressure drop measured at 30℃

The symbol "-" indicates either absence of adsorbent or material washout by mobile phase, precluding further experiments.

TABLE S2 Fractions obtained from F. tikoua under different mobile phase conditions

| Enrichment mobile phase | Non-retained components | Retained components |
| --- | --- | --- |
| Methanol:water (60:40, v/v) | F1 | F2 |
| Methanol:water (50:50, v/v) | F3 | F4 |
| Methanol:water (40:60, v/v) | F5 | F6 |
| Methanol:water (30:70, v/v) | F7 | F8 |
| Methanol:water (20:80, v/v) | F9 | F10 |
| Methanol:water (10:90, v/v) | F11 | F12 |

TABLE S3 Gradient elution parameters for secondary separation of *Ficus tikoua Bur.* fractions

| Time (min) | Flow rate (mL/min) | Acetonitrile content (%)· | Water content (%) |
| --- | --- | --- | --- |
| 0 | 1 | 60 | 40 |
| 10 | 2 | 64 | 36 |
| 25 | 2 | 65 | 35 |
| 30 | 2 | 70 | 30 |
| 35 | 2 | 75 | 25 |
| 65 | 2 | 95 | 5 |
| 80  90  110  130 | 2  2  2  1 | 100  100  80  60 | 0  0  20  40 |
